# Supplementary material for: Effective CRISPRa-mediated control of gene expression in bacteria must overcome strict target site requirements
Source: Nat Commun. 2020 Apr 1;11:1618. doi: 10.1038/s41467-020-15454-y (PMC7113249; doi:10.1038/s41467-020-15454-y)
Supplement: Supplementary file 1 — Supplementary Information [file 41467_2020_15454_MOESM1_ESM.pdf]

Effective CRISPRa-mediated control of gene expression in bacteria must overcome strict target site requirements

Fontana and Dong *et al.*

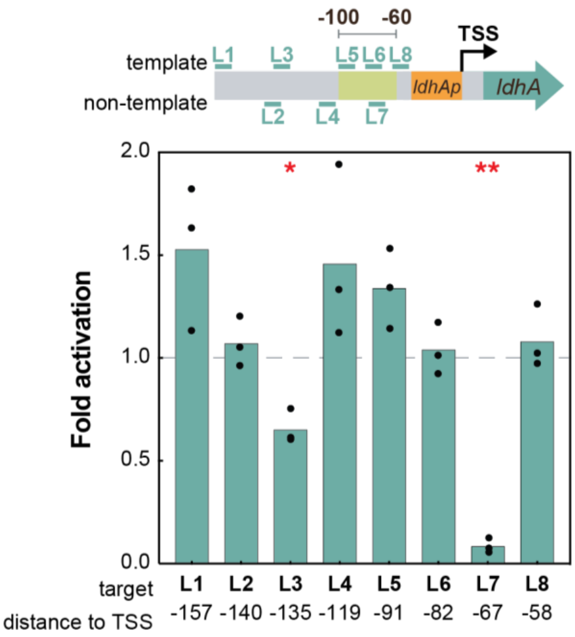

Supplementary Figure 1. CRISPRa at the endogenous gene target *ldhA* does not follow predicted trends.

Eight scRNA target sites (L1-L8) upstream of the *ldhA* promoter were selected. Three of the target sites (L5-L7) were within the 40 bp window where CRISPRa is effective (-100 to -60). While L1, L4, and L5 resulted in weak increases in gene expression, there was no apparent relationship between the position of the sites and *ldhA* expression levels. Gene expression was measured using RT-qPCR. Fold activation represents expression levels relative to an off-target control (hAAVS1). Values represent the average calculated from n = 3 technical replicates. Stars indicate a statistically significant difference from the off-target control using a two-tailed unpaired Welch's t-test (*: *p-value* < 0.05, **: *p-value* < 0.01). Exact *p-values*: L1: 0.12, L2: 0.43, L3: 0.02, L4: 0.20, L5: 0.09, L6: 0.67, L7: 0.0005, L8: 0.45. Source data are provided as a Source Data file.

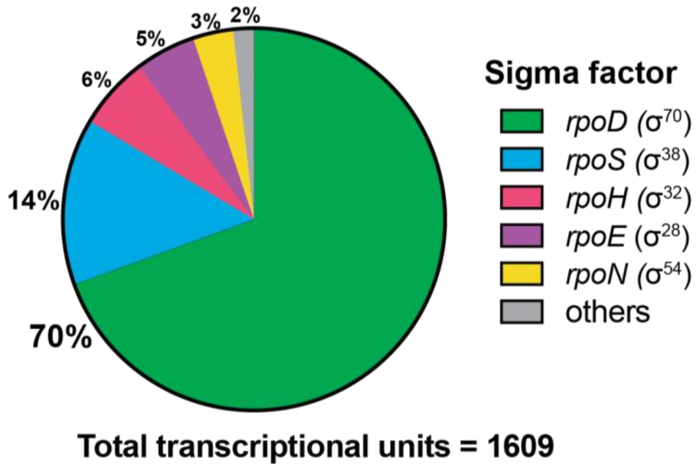

Supplementary Figure 2. Distribution of transcriptional units regulated by sigma factors. The number of *E. coli* transcriptional units regulated by sigma factors was obtained from Ecocyc[1] on the “Regulon” tab of the page relative to each sigma factor. The total number of transcriptional units represents the sum of the transcriptional units regulated by each sigma factor. Source data are provided as a Source Data file.

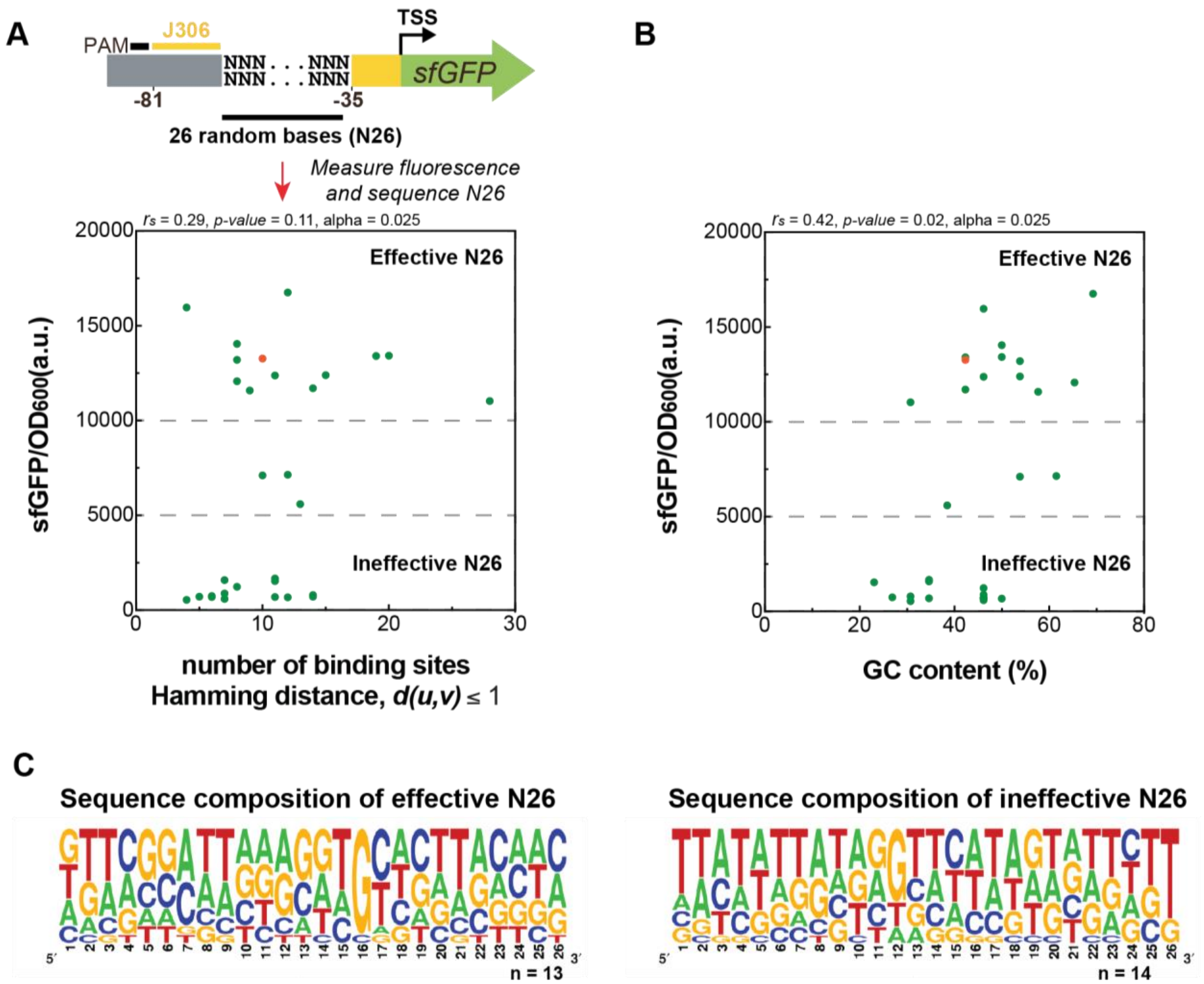

Supplementary Figure 3. Intervening sequences between the scRNA target site and minimal promoter that interfere with CRISPRa tend to be more AT-rich.

A) Plot of gene activation vs. transcription factor binding sites present in the intervening sequence between the scRNA target site and the minimal promoter. CRISPRa was targeted to a reporter library with 26 randomized bases between the scRNA target site and the -35 region on the J3-J23117-sfGFP reporter (N26), and we observed a 27-fold variation in gene activation (Figure 3C). We sequenced 29 variants and identified a number of motifs within one base of known consensus transcription factor binding (Supplementary Table 6). In parallel, we measured gene activation (sfGFP/OD_{600}) for each strain. The plot shows the sfGFP/OD_{600} of each strain versus the number of sequences within a Hamming distance $d(u,v) \leq 1$ from the consensus sequences of transcription factor binding sites, obtained from RegulonDB[2]. r_s indicates the Spearman rank order correlation coefficient between sfGFP/OD_{600} and number of binding sites, and its associated two-tailed *p-value* is relative to the null hypothesis of no correlation between sfGFP/OD_{600} and number of binding sites, with a Bonferroni-corrected α =

0.025. Green dots indicate the sfGFP/OD_{600} values and the number of binding sites calculated from individual colonies, and the orange dot indicates a strain where CRISPRa is targeting the original J3-J23117-sfGFP promoter. **B)** Plot of gene activation vs. GC content in the intervening sequence between the scRNA target site and the minimal promoter. r_s indicates the Spearman rank order correlation coefficient between sfGFP/OD_{600} and GC content, and its associated two-tailed *p-value* is relative to the null hypothesis of no correlation between sfGFP/OD_{600} and GC content, with a Bonferroni-corrected $\alpha = 0.025$. In panels A and B, green dots indicate the sfGFP/OD_{600} values and GC content calculated from n = 1 biologically independent samples, and the orange dot indicates a strain where CRISPRa targets the original J3-J23117-sfGFP promoter. **C)** Logo plots (https://weblogo.berkeley.edu/) showing the base composition of N26 sequences effective for CRISPRa (sfGFP/OD_{600} > 10000 a.u., 13 sequences) and N26 sequences ineffective for CRISPRa (sfGFP/OD_{600} < 5000 a.u., 14 sequences). Source data of Supplementary Figure 3A and 3B are provided as a Source Data file.

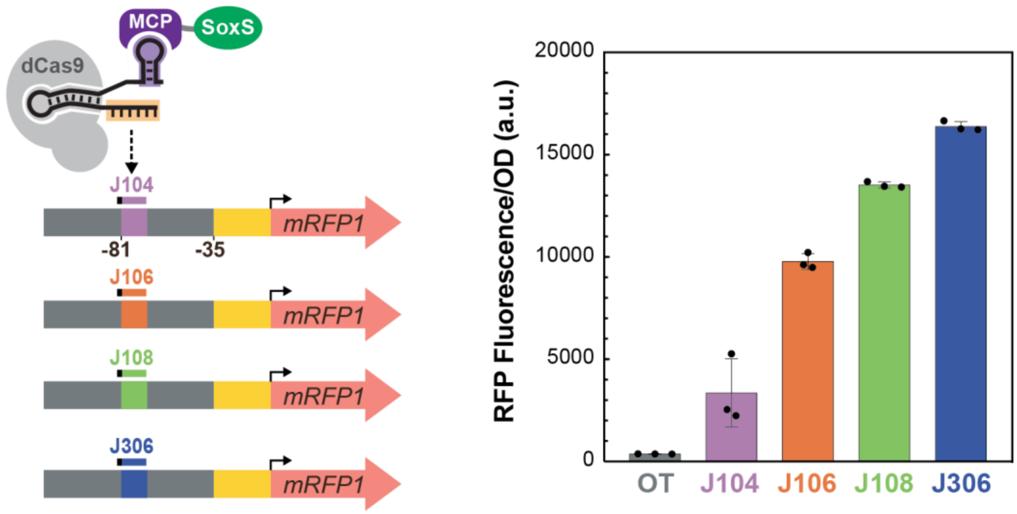

Supplementary Figure 4. CRISPRa activity depends on the target sequence on the scRNA.

Reporter cassettes that differ only by the sequence of the 20 base scRNA target site give a broad range of gene expression levels, demonstrating that the sequence of the scRNA target site can have a substantial effect on CRISPRa. Three new reporter plasmids were constructed where the J306 target site, located at -81 from the TSS, on the J3-J23117-mRFP1 reporter was replaced by the J104, J106, and J108 sequence. Activation at each promoter was tested when CRISPRa was targeted to their cognate scRNA site. The off-target negative control (OT) represents a strain expressing the original reporter with the J306 site and the CRISPRa components to target an off-target site (J206). Values represent the average +/- standard deviation calculated from n = 3 biologically independent samples. Source data are provided as a Source Data file.

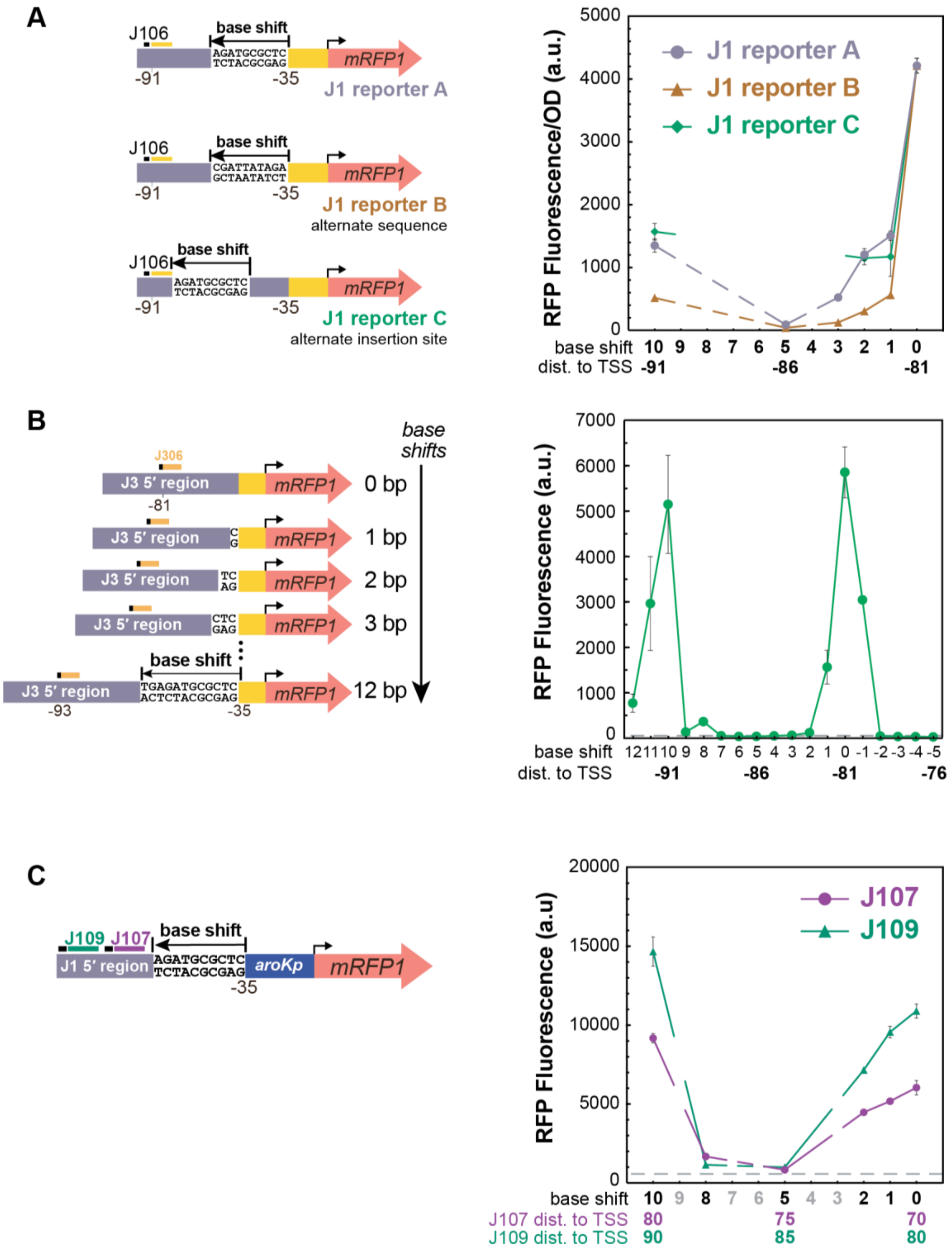
A
J106
base shift
AGATGCGCTC
TCTACGCGAG
mRFP1
-91
-35
J1 reporter A
J106
base shift
CGATTATAGA
GCTAATATCT
mRFP1
-91
-35
J1 reporter B
alternate sequence
base shift
J106
AGATGCGCTC
TCTACGCGAG
mRFP1
-91
-35
J1 reporter C
alternate insertion site
J1 reporter A
J1 reporter B
J1 reporter C
RFP Fluorescence/OD (a.u.)
base shift 10 9 8 7 6 5 4 3 2 1 0
dist. to TSS -91 -86 -81
B
base shifts
J306
J3 5′ region
mRFP1
0 bp
-81
J3 5′ region
mRFP1
1 bp
J3 5′ region
mRFP1
2 bp
J3 5′ region
mRFP1
3 bp
base shift
J3 5′ region
TGAGATGCGCTC
ACTCTACGCGAG
mRFP1
12 bp
-93
-35
RFP Fluorescence (a.u.)
base shift 12 11 10 9 8 7 6 5 4 3 2 1 0 -1 -2 -3 -4 -5
dist. to TSS -91 -86 -81 -76
C
J109
J107
base shift
J1 5′ region
AGATGCGCTC
TCTACGCGAG
aroKp
mRFP1
-35
J107
J109
RFP Fluorescence (a.u)
base shift 10 9 8 7 6 5 4 3 2 1 0
J107 dist. to TSS 80 75 70
J109 dist. to TSS 90 85 80

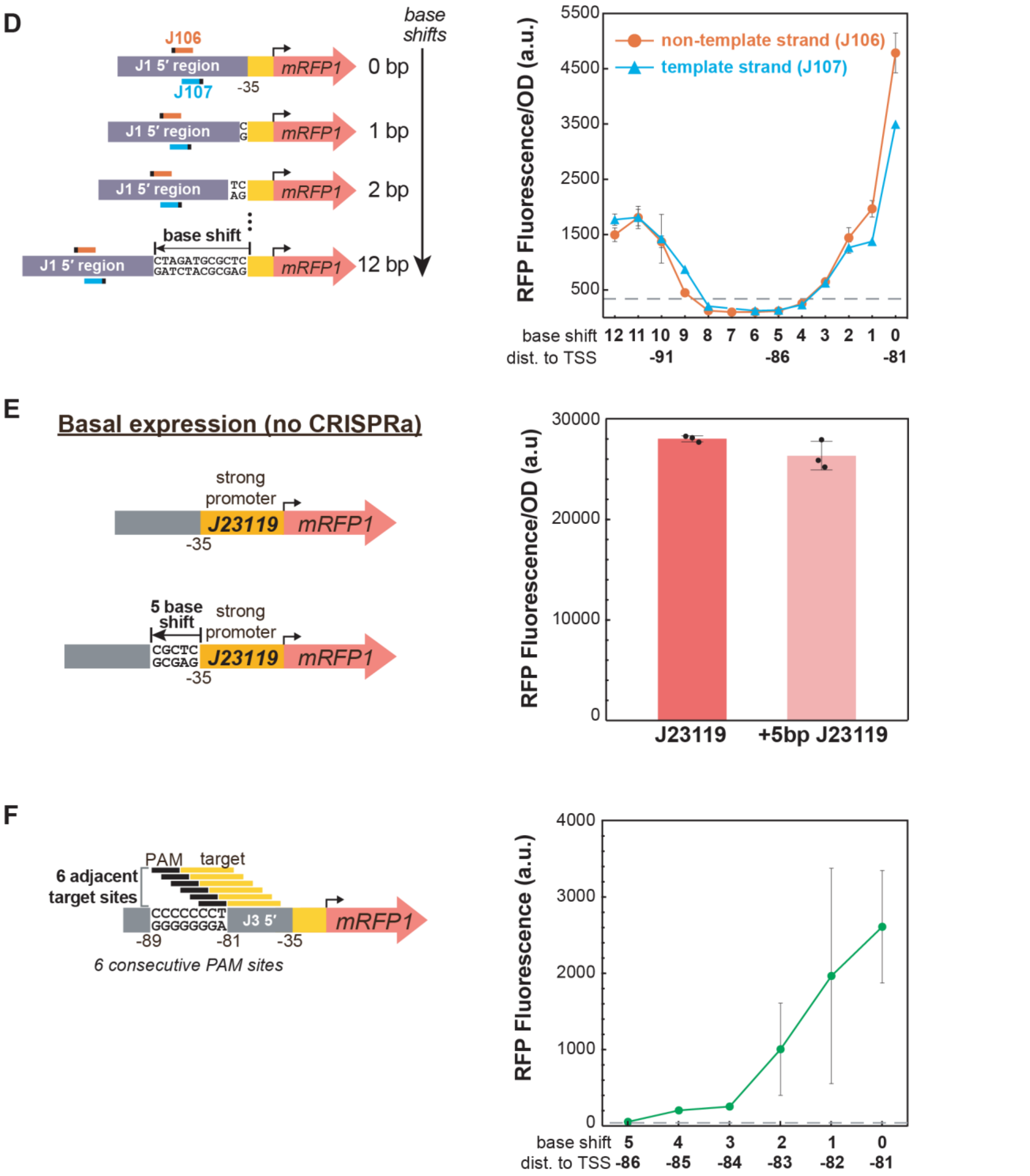

Supplementary Figure 5. The sharp positioning dependence of CRISPRa is observed across multiple promoters.

A) The sharp positioning requirements of CRISPRa are not significantly affected by the location or composition of the inserted sequence. Reporters were based on the J1-J23117-mRFP1 (Figure 4) with base shifts introduced in different ways. In the J1 reporter A, bases were inserted upstream of the -35 region. In the J1 reporter B, a different sequence was inserted at the same

site. In the J1 reporter C, bases were inserted downstream of the J106 target site. There were modest differences between reporter A and reporter B that could indicate a contribution from sequence composition. **B)** The sharp positioning requirements of CRISPRa were observed with a different heterologous promoter. CRISPRa was targeted at the -81 site on the J3-J23117 promoter that has a different upstream sequence and a different scRNA target site (J306) compared to J1-J23117. Peaks in gene expression were observed at -81 and -91 to the TSS, displaying a 10 bp periodicity. The peaks of gene expression on the J3-J23117 promoter were sharper than in the J1-J23117 promoter (Figure 4A); gene expression decreased to baseline levels after shifting only 2 bp from the peak position. After a complete 10 bp-shift period, CRISPRa activity was fully restored to the original peak expression. Reporter gene sets were constructed by inserting 0-12 bp or deleting 1-5 bp upstream of the -35 of the J3-J23117-mRFP1 reporter. The grey line represents the baseline activity of the J3-J23117-mRFP1 reporter strain containing an empty vector instead of the CRISPRa component plasmid. For comparison, previous CRISPRa data at -81 and -91 are shown on the schematic above the plot[3]. **C)** The sharp positioning requirements of CRISPRa are observed when targeting a different minimal promoter. The J1-*aroKp2* promoter displayed positioning requirements similar to the J1-J23117 promoter. Decreases in CRISPRa activity after 3 bp shifts and recovery at a 10 bp shift were observed on both the J107 and the J109 target sites. The J1-*aroKp2* promoter was constructed by replacing the BBa_J23117 minimal promoter from the J1-J23117 promoter to the aroKp2 minimal promoter. A J1-*aroKp2*-mRFP1 reporter series was constructed by adding 0 bp, 1 bp, 2 bp, 5 bp, 8 bp, and 10 bp upstream of the -35 region. The grey line represents the baseline activity of the J1-*aroKp2*-mRFP1 reporter reporter strain expressing a CRISPRa component plasmid with an off-target scRNA. **D)** The sharp positioning requirements of CRISPRa are observed on both the template and the non-template strand. For both the J106 target site on the non-template strand and the J107 target on the template strand, the CRISPRa activity decreased to baseline levels after shifting 3 bp from its original position and recovered after shifting 10 bp. The reporter plasmid was the same as in Figure 4A. The dotted grey line indicates the negative control where an off-target scRNA (J206) was co-transformed with the original reporter with no inserted bases. **E)** Adding 5 bases adjacent to the -35 region does not dramatically alter the expression of the promoter. The 5 bases added were the same as those used to shift the J1 and J3 promoters (Figure 4A and Supplementary Figure 5B). This experiment was performed using a strong minimal promoter (BBa_J23119), so that any detrimental effects would be detectable. **F)** The sharp positioning requirements of CRISPRa were observed when tested in a single reporter with multiple consecutive PAM sites. 6

consecutive PAM sites on the non-template strand were introduced by placing a CCCCCCCT sequence between -89 and -81 bp to the TSS on the J3-J23117-mRFP1 reporter. Maximum gene expression was observed at the original -81 site, after which expression gradually decreased to one third of the maximum activity after moving 2 bp away (-83). Gene expression decreased further when the scRNA target was moved 3 bp and 4 bp away from the TSS (-84 and -85), and reached the baseline when moved 5 bp (-86). The grey line represents the baseline activity of the reporter strain containing an empty vector instead of the CRISPRa component plasmid. Values in panels A-F represent the average +/- standard deviation calculated from n = 3 biologically independent samples. Source data are provided as a Source Data file.

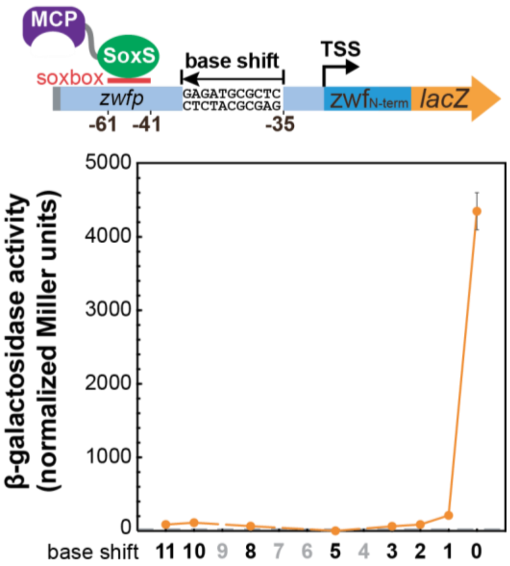

Supplementary Figure 6. Wild type SoxS displays a sharp positioning requirement when targeting a SoxS-dependent promoter.

When the wild type SoxS binding site (soxbox) on the endogenous *zwfp* promoter is shifted by 1 bp, gene expression decreases significantly, consistent with previous reports[4]. Shifting the soxbox further upstream causes gene expression to completely reduce to the baseline. Reporter plasmids were constructed by adding 1, 2, 3, 5, 8, 10, and 11 bp upstream of the -35 on the *zwfp-lacZ* reporter (Figure 1). Values represent the average β-galactosidase activity in normalized Miller +/- standard deviation calculated from n = 3 biologically independent samples. Source data are provided as a Source Data file.

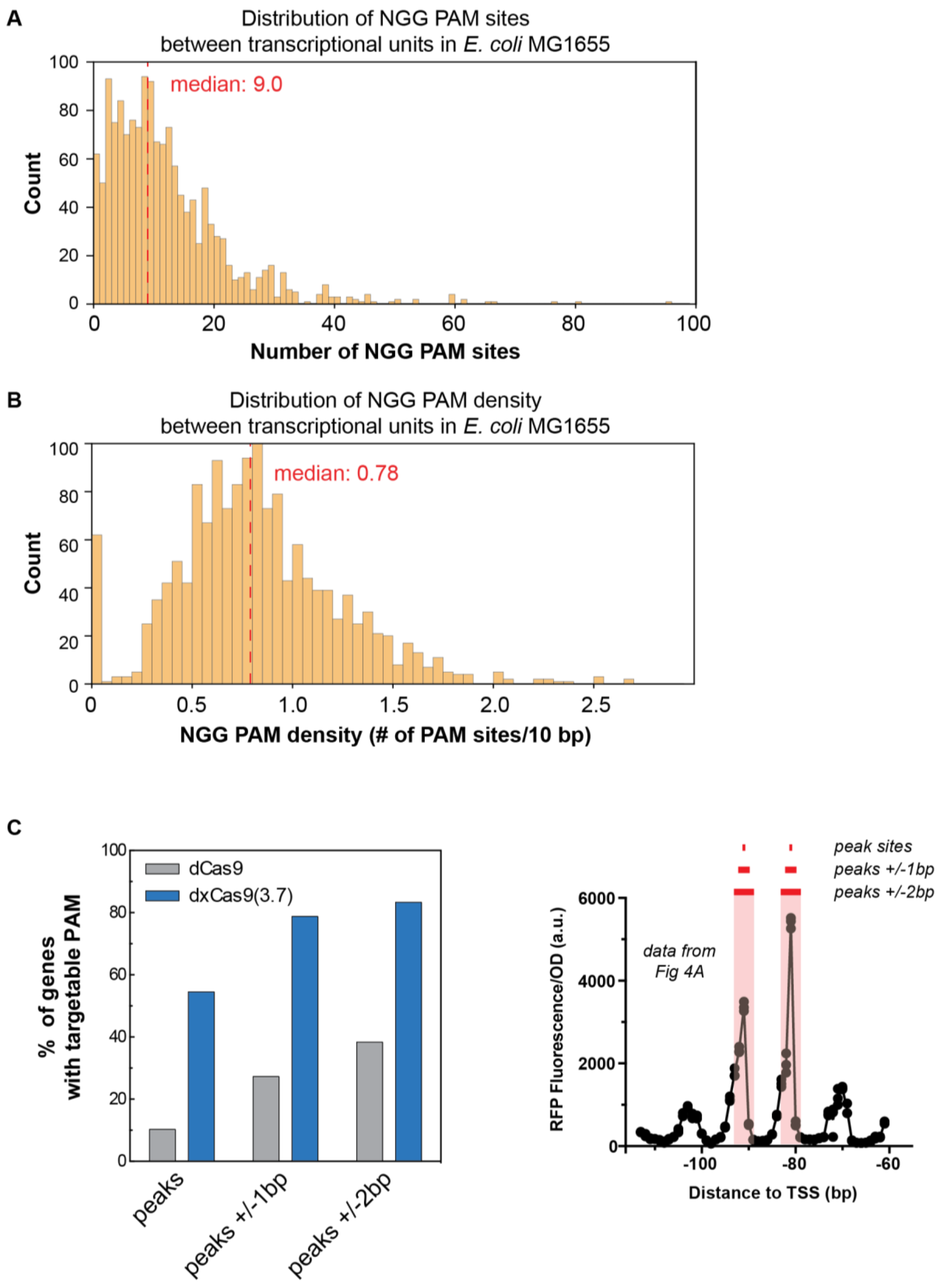

Supplementary Figure 7. Availability of PAM sites between transcriptional units in *E. coli* MG1655.

A) Distribution of the number of NGG PAM sites between transcriptional units in *E. coli*

MG1655. The median number of NGG PAM sites between transcriptional units in *E. coli* MG1655 is 9.0. Methods for extracting the sequence of the DNA regions between transcriptional units in *E. coli* MG1655 and identifying available PAM sites are described in the Supplementary Methods.

B) Distribution of the density of NGG PAM sites between transcriptional units in *E. coli* MG1655. The density of PAM sites is reported over a 10 bp window. This window size was chosen because it corresponds to a full turn of the DNA helix. The density of PAM sites in a 10 bp window was calculated for each sequence between transcriptional units as the total number of PAM sites in the sequence divided by the length of the intergenic sequence and then multiplied by 10. The median density of NGG PAM sites per 10 bp between transcriptional units in *E. coli* MG1655 is 0.78. **C)** The number of genes in the *E.coli* genome that can be targeted by dCas9 is small, and the expanded PAM variant dxCas9(3.7) significantly increases the number of genes with predicted effective target sites for CRISPRa. The plot to the left shows percentage of genes with at least one PAM site targetable by dCas9 or dxCas9(3.7) at: the positions where CRISPRa displays a peak in activity. The plot to the right illustrates the range of positions on the non-template strand chosen for the analysis. Corresponding peaks on the template strand were also included. Analyses were performed using data generated when selecting candidate endogenous genes for activation (Supplementary Methods). Source data are provided as a Source Data file.

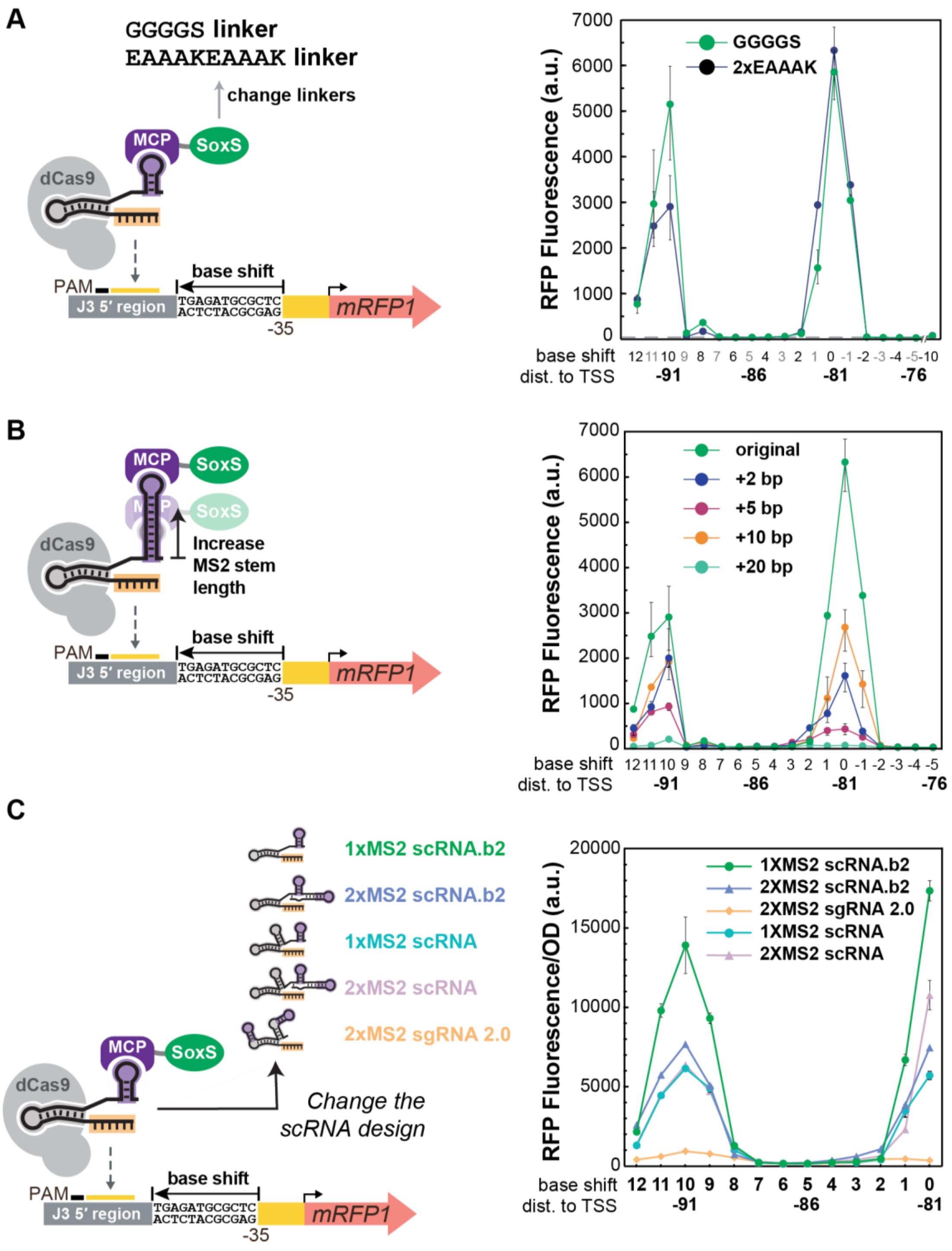

Supplementary Figure 8. Modifying the CRISPRa complex structure does relax the sharp positioning requirements of CRISPRa.

A) Changing the linker between MCP and SoxS does not change the positioning dependence of CRISPRa. A CRISPRa complex with the MCP-(EAAAKEAAAK)-SoxS(R93A/S101A) activation domain displayed 10 bp periodicity of peak expression similar to that with the MCP-(GGGGS)-SoxS(R93A/S101A) activation domain. The EAAAKEAAAK linker is predicted to be more rigid than the GGGGS linker[5]. The grey line represents the baseline activity of the J3-J23117-mRFP1 reporter strain containing an empty vector instead of the CRISPRa component plasmid. **B)** Extending the length of the MS2 stem does not change the positioning dependence of CRISPRa. CRISPRa systems with scRNAs that have +2, +5, and +10 RNA base pairs added to the bottom of the MS2 stem displayed the same 10 bp periodicity but lower peak activity compared to the original 1xMS2 scRNA.b2. The strain having the scRNA with +20 bp extended MS2 stem did not show any CRISPRa activity. **C)** No improvements in the effective target range were observed for CRISPRa systems with alternative scRNA designs. The scRNA designs tested are: 1xMS2 scRNA.b2 and 2XMS2 scRNA.b2 with the tracrRNA hairpin removed[3], the original 1xMS2 and 2XMS2 scRNA design having the tracrRNA hairpin[6], and sgRNA 2.0 where the MS2 hairpins are extended from the RNA stems on the sgRNA[7]. CRISPRa systems expressing a 2XMS2 scRNA.b2, 1xMS2 scRNA, and 2XMS2 scRNA displayed the same 10 bp periodicity but lower peak activity than 1xMS2 scRNA.b2. Expressing a sgRNA 2.0 resulted in no CRISPRa activity. Values in panels A-C represent the average +/- standard deviation calculated from n = 3 biologically independent samples. Source data are provided as a Source Data file.

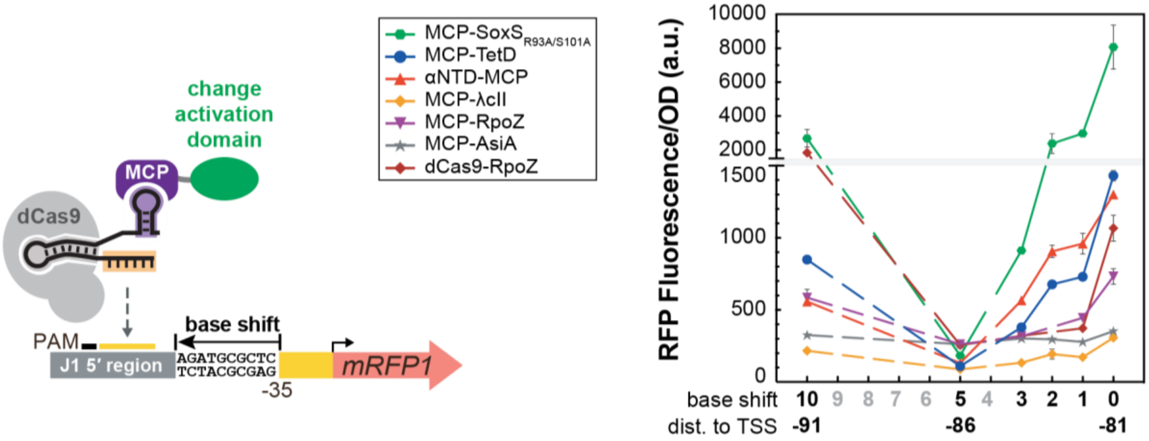

Supplementary Figure 9. Performing CRISPRa with alternative activation domains does not expand the range of targetable positions.

CRISPRa with MCP-TetD and αNTD-MCP activation domains[3] displayed similar positioning dependence as MCP-SoxS(R93A/S101A), where gene expression decreases as the position shifts from 0 to 3 bp, reaches the baseline at a 5 bp shift, and increases again at a 10 bp shift. CRISPRa with MCP-RpoZ[3] and dCas9-RpoZ[8] activation domains displayed more stringent positioning dependence, where gene expression approaches the baseline at 1 bp shift. All alternative activation domains gave weaker peak gene expression at 0 bp shift compared to MCP-SoxS(R93A/S101A). MCP-λcII and MCP-AsiA[3] activation domains did not show any significant CRISPRa activity. All activation domains were cloned into the CRISPRa component plasmid containing *Sp*-dCas9 and 1xMS2 scRNA.b2 targeting J106. To reduce the toxicity of AsiA, the MCP-AsiA plasmid also contains a co-expressed *rpoD(F563Y)* gene[3]. The MCP-SoxS(R93A/S101A), MCP-TetD, αNTD-MCP, MCP-λcII and MCP-AsiA plasmids were tested in *E. coli* MG1655. The MCP-*rpoZ* and dCas9-*rpoZ* plasmids were tested in the *E. coli* strain CD03 (MG1655/Δ*rpoZ*) (Supplementary Table 1)[3]. Values represent the average +/- standard deviation calculated from n = 3 biologically independent samples. Source data are provided as a Source Data file.

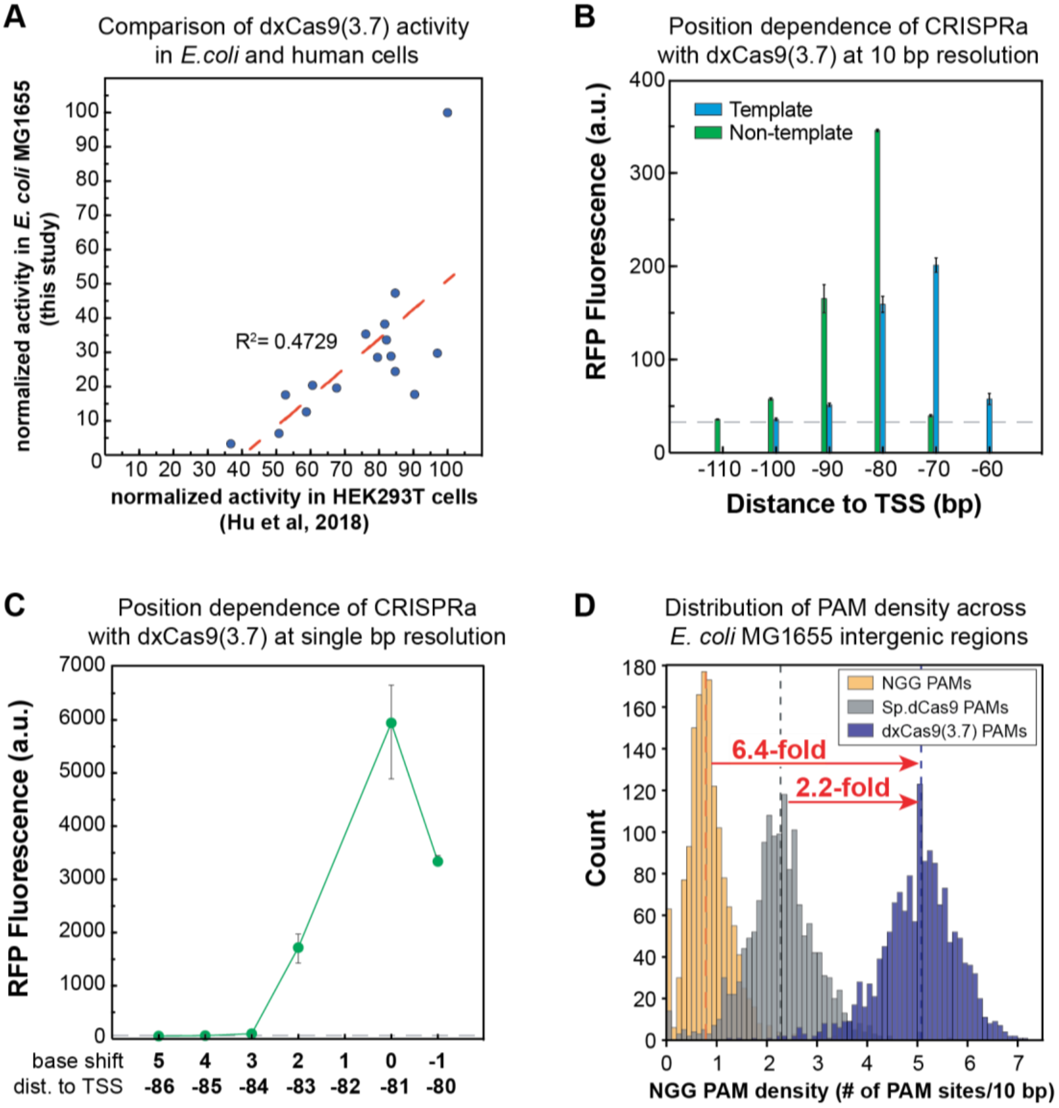

Supplementary Figure 10. dxCas9(3.7) can target an expanded range of PAM sites and is sensitive to target site position for CRISPRa.

A) Relative CRISPRa activities of dxCas9(3.7) on different PAM targets in *E. coli* correlates well with the corresponding data obtained by Hu et al.[9] in human cells. The CRISPRa activity on the NGG PAM site in both studies were normalized to 100 and the CRISPRa activity on all the other PAM sites were normalized to the value of NGG PAM site. Normalized data in this study (y axis) were plotted against the normalized data obtained by Hu et al. (x axis). **B)** CRISPRa with dxCas9(3.7) displayed similar sensitivity to target site position as *Sp*-dCas9. Peaks of activation were observed at -81 and -91 on the non-template strand and -70 and -80 on the template strand. CRISPRa was targeted to a J1-J23117-mRFP1 reporter integrated into the genome (*E. coli* CD13, Supplementary Table 1). Values represent the average +/- standard deviation

calculated from n = 3 biologically independent samples. The grey dotted line represents the baseline fluorescence of a strain containing the dxCas9(3.7) and MCP-SoxS(R93A/S101A) and an empty vector with no scRNAs. **C)** CRISPRa with dxCas9(3.7) displayed similar positioning dependence with single base shifts. Gene expression was significantly reduced when the scRNA target site was shifted 1-2 bp in either directions from the optimal position -81 bp to the TSS on the non-template strand. Shifting the scRNA target site 3-5 bp causes gene expression to fall to the baseline level. Reporter gene sets were constructed with 1 bp deleted or 2-5 bp inserted upstream of the -35 of the J3-J23117-mRFP1 reporter. The grey line represents the baseline activity of a strain containing the J3-J23117-mRFP1 reporter plasmid and a CRISPRa component plasmid with an off-target scRNA (hAAVS1). Values represent the average +/- standard deviation calculated from n = 3 biologically independent samples. **D)** dxCas9(3.7) increases the probability of finding targetable PAM sites for CRISPRa. Histograms showing the distribution of the PAM density (defined in Supplementary Figure 7B) between transcriptional units in *E. coli* MG1655 suggest that the average likelihood of finding a dxCas9(3.7)-compatible PAM (blue) is ~6.4-times higher than finding an NGG PAM (yellow), and ~2.2-times higher than finding a *Sp*-dCas9-compatible PAM (grey). Vertical dotted lines indicate the median PAM density for each group. The median PAM density is 5.08 for dxCas9(3.7)-compatible PAMs (NGG, AGA, AGC, AGT, CGA, CGC, CGT, GGA, GGC, GGT, TGA, TGC, TGT, GAA, GAT, CAA), 2.33 for *Sp*-dCas9-compatible PAMs (NGG, AGA, CGA, GGA, GGC, GGT, TGA). and 0.78 for NGG PAMs. Methods for extracting the sequence of *E. coli* MG1655 intergenic regions, counting targetable PAM sites and calculating the PAM density are described in the Supplementary Methods. Source data are provided as a Source Data file.

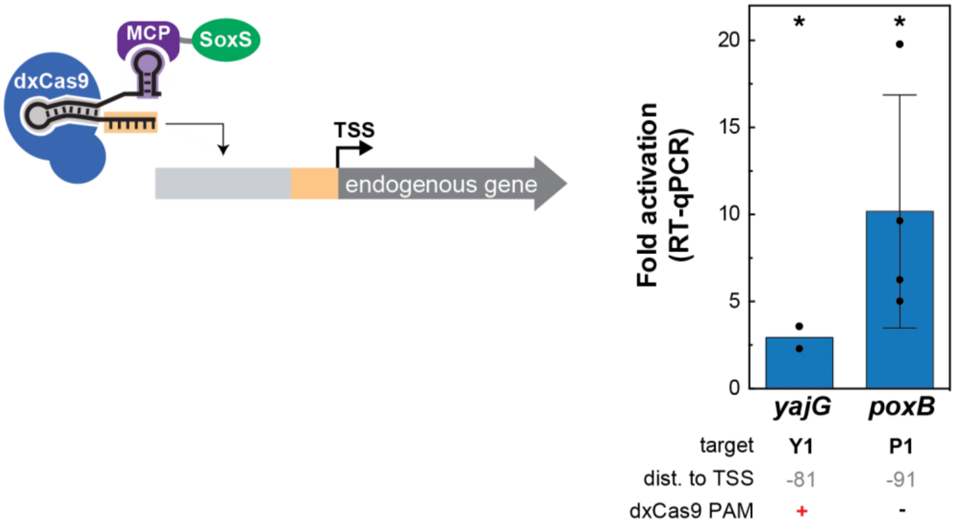

Supplementary Figure 11. Predictive rules for CRISPRa enable activation of endogenous genes *yajG* and *poxB*.

The scRNA sites displaying the highest activity on the *yajG* and *poxB* reporters from the *E. coli* promoter collection[10] (Figure 6B) were tested for activity with dxCas9(3.7) at the endogenous *yajG* and *poxB* genes using RT-qPCR. Fold activation represents expression levels relative to a control expressing an off-target scRNA (J306). For Y1, the value represents the average calculated from n = 2 biologically independent samples. For P1, the value represents the average +/- standard deviation calculated from n = 3 biologically independent samples. The (*) symbol indicates a statistically significant difference from the off-target control (*p-value* < 0.05 using a two-tailed unpaired Welch's t-test). Exact *p-values*: Y1: 0.02, P1: 0.04. Source data are provided as a Source Data file.

Supplementary Table 1. *E. coli* strains.

**Strain**	**Description**	**Genotype**	**Reference**
MG1655	parent *E. coli* strain	F- λ- ilvG- rfb-50 rph-1	
CD03	MG1655 with *rpoZ* knocked out	MG1655 Δ*rpoZ*	[3]
CD06	MG1655/sfGFP (weak promoter)	MG1655 *W1-BBa_J23117-sfGFP KanR::nfsA*	[3]
CD13	MG1655/mRFP1 (weak promoter)	MG1655 *J1-BBa_J23117-mRFP1::nfsA*	This study, Suppl. Fig. 8

Supplementary Table 2. Description of the CRISPRa systems used in each figure.

Figure	Cas protein	scRNA design	scRNA target	Activation domain
1B	dCas9	1xMS2 scRNA.b1	W108	MCP-(5aa)-SoxS(wild-type or mutant)
2A	dCas9	1xMS2 scRNA.b1	A1-A4, hAAVS1	MCP-(5aa)-SoxS(R93A/S101A)
2B	dCas9	1xMS2 scRNA.b2	C1-C5, hAAVS1	MCP-(5aa)-SoxS(R93A)
3A	dCas9	1xMS2 scRNA.b2	J306, J206	MCP-(5aa)-SoxS(R93A)
3B	dCas9	1xMS2 scRNA.b2	J101-J120, hAAVS1	MCP-(5aa)-SoxS(R93A/S101A)
3C	dCas9	1xMS2 scRNA.b2	J306, J206	MCP-(5aa)-SoxS(R93A)
3D	dCas9	1xMS2 scRNA.b2	J306, J206	MCP-(5aa)-SoxS(R93A/S101A)
4A	dCas9	1xMS2 scRNA.b2	J102, J104, J106, J108, J110, J206	MCP-(5aa)-SoxS(R93A)
4B	dCas9	1xMS2 scRNA.b2	J106	MCP-(5aa)-SoxS(R93A), MCP-(10aa)-SoxS(R93A), MCP-(20aa)-SoxS(R93A)
5A	dCas9 / dxCas9 (3.7)	1xMS2 scRNA.b2	J306	MCP-(5aa)-SoxS(R93A/S101A)
5B	dCas9 / dxCas9 (3.7)	1xMS2 scRNA.b2	M1, M2, J206	MCP-(5aa)-SoxS(R93A/S101A)
6A	dCas9 / dxCas9 (3.7)	1xMS2 scRNA.b2	Y1-Y3, J306	MCP-(5aa)-SoxS(R93A/S101A), tet-inducible
6B	dxCas9	1xMS2	Y1, Y2, P1, P2,	MCP-(5aa)-

	(3.7)	scRNA.b2	U1, U2, D1, D2, B1, B2, E1, E2, J306	SoxS(R93A/S101A), tet-inducible
Supplementary Figure 1	dCas9	1xMS2 scRNA.b2	L1-L8, hAAVS1	MCP-(5aa)-SoxS(R93A)
Supplementary Figure 3A	dCas9	1xMS2 scRNA.b2	J306, J206	MCP-(5aa)-SoxS(R93A)
Supplementary Figure 4	dCas9	1xMS2 scRNA.b2	J104, J106, J108, J306, J206	MCP-(5aa)-SoxS(R93A)
Supplementary Figure 5A	dCas9	1xMS2 scRNA.b2	J106	MCP-(5aa)-SoxS(R93A)
Supplementary Figure 5B	dCas9	1xMS2 scRNA.b2	J306	MCP-(5aa)-SoxS(R93A/S101A)
Supplementary Figure 5C	dCas9	1xMS2 scRNA.b2	J107, J109	MCP-(5aa)-SoxS(R93A/S101A)
Supplementary Figure 5D	dCas9	1xMS2 scRNA.b2	J106, J107, J206	MCP-(5aa)-SoxS(R93A)
Supplementary Figure 5F	dCas9	1xMS2 scRNA.b2	J306, J306+1-5	MCP-(5aa)-SoxS(R93A/S101A)
Supplementary Figure 8A	dCas9	1xMS2 scRNA.b2	J306	MCP-(5aa)-SoxS(R93A/S101A), MCP-(2xEAAAK)-SoxS(R93A/S101A)
Supplementary Figure 8B	dCas9	1xMS2 scRNA.b2, 1xMS2 scRNA.b2 + 2/5/10 bp MS2 stem extension	J306	MCP-(2xEAAAK)-SoxS(R93A/S101A)
Supplementary Figure 8C	dCas9	1xMS2 scRNA.b2, 2xMS2	J306	MCP-(5aa)-SoxS(R93A)

		scRNA.b2, 2xMS2 sgRNA2.0, 1xMS2 scRNA, 2xMS2 scRNA		
Supplementary Figure 9	dCas9	1xMS2 scRNA.b2	J106	MCP-(5aa)-SoxS(R93A/S101A), Alternative activators
Supplementary Figure 10B	dxCas9 (3.7)	1xMS2 scRNA.b2	J104-J113	MCP-(5aa)-SoxS(R93A)
Supplementary Figure 10C	dxCas9 (3.7)	1xMS2 scRNA.b2	J306	MCP-(5aa)-SoxS(R93A)
Supplementary Figure 11	dxCas9 (3.7)	1xMS2 scRNA.b2	Y1, P1, J306	MCP-(5aa)-SoxS(R93A/S101A), tet-inducible

Supplementary Table 3. gRNA target sites.

sgRNA target	DNA Sequence	Target Strand[a]	Distance to TSS[b]
W108	GAAGATCCGGCCTGCAGCCA	NT	91
J101[c]	TGGGTTCCACCGGATACCTC	T	40
J103[c]	AGGCGTCCTTTGGGTTCCAC	T	50
J105[c]	CGGTTACCAAAGGCGTCCTT	T	60
J107[c]	CGGTGTCCTGCGGTTACCAA	T	70
J109[c]	AGGTATCCTGCGGTGTCCTG	T	80
J111[c]	GGGCGACCTCAGGTATCCTG	T	90
J113[c]	GGGCCACCACGGGCGACCTC	T	100
J115[c]	TGGTGACCATGGGCCACCAC	T	110
J117[c]	GGGTGACCTATGGTGACCAT	T	120
J119[c]	TGGTTGCCAAGGGTGACCTA	T	130
J121[c]	AGGACACCTTTGGTTGCCAA	T	140
J102[c]	AGGTATCCGGTGGAACCCAA	NT	61
J104[c]	TGGAACCCAAAGGACGCCTT	NT	71
J106[c]	AGGACGCCTTTGGTAACCGC	NT	81
J108[c]	TGGTAACCGCAGGACACCGC	NT	91
J110[c]	AGGACACCGCAGGATACCTG	NT	101
J112[c]	AGGATACCTGAGGTCGCCCG	NT	111
J114[c]	AGGTCGCCCGTGGTGGCCCA	NT	121
J116[c]	TGGTGGCCCATGGTCACCAT	NT	131
J118[c]	TGGTCACCATAGGTCACCCT	NT	141
J120[c]	AGGTCACCCTTGGCAACCAA	NT	151
hAAVS1[c]	GGGGCCACTAGGGACAGGAT	off-target	n/a
J206	TAGTAGCCGAACACGTCCTC	off-target	n/a
J306	TTGTGTCCAGAACGCTCCGT	NT	81
J306+1	TGTGTCCAGAACGCTCCGTA	NT	82
J306+2	GTGTCCAGAACGCTCCGTAG	NT	83
J306+3	TGTCCAGAACGCTCCGTAGG	NT	84
J306+4	GTCCAGAACGCTCCGTAGGG	NT	85
J306+5	TCCAGAACGCTCCGTAGGGG	NT	86
M1	AGCAGAAGTGTCAGCAGTGT	NT	81 (reporter A), 86

			(reporter B)
M2	CGACGAGCAGAAGTGTCAGC	NT	76 (reporter A), 81 (reporter B)
aroKB_A1	GGGCAATTATTTCGTCATGA	T	151
aroKB_A2	AGATGAACGACGCGAGTTAG	T	122
aroKB_A3	TTTTACGGCTGTTTACTCAC	NT	92
aroKB_A4	TGAGTAAACAGCCGTAAAAG	T	71
cysK_C1	CCACCCCTGTTTCACACAAA	NT	93
cysK_C2	AAACCGTTTGTGTGAAACAG	T	79
cysK_C3	GACATGCAAGATGGAATAAG	NT	61
cysK_C4	ATGACATGCAAGATGGAATA	NT	59
cysK_C5	GGAAATAATGACATGCAAGA	NT	52
ldhA_L1	CAGTAATAACAGCGCGAGAA	T	157
ldhA_L2	GGATATTAACTACCCATGCT	NT	140
ldhA_L3	GCTTTATATTTACCCAGCAT	T	135
ldhA_L4	GCTTAATTTTTCGCTAAATC	NT	119
ldhA_L5	GAAAAATTAAGCATTCAATA	T	91
ldhA_L6	AGCATTCAATACGGGTATTG	T	82
ldhA_L7	AGGCGCAACCTTCAACTGAA	NT	67
ldhA_L8	ATGTTTAACCGTTCAGTTGA	T	58
yajG_Y1	TTGACGAAATAATCGCCCCT	NT	81
yajG_Y2	CATCAGTGTTTCTTTTACCA	T	80
yajG_Y3	AAATAATCGCCCCTGGTAAA	NT	87
poxB_P1	CCCGATGAAAGGAATATCAT	NT	91
poxB_P2	GGTTAAATAGCCCGATGAAA	NT	81
uxuR_U1	TGATTGACCAGTAAGTCTGT	NT	81
uxuR_U2	GATTACCCTACAGACTTACT	T	70
ppiD_D1	ACTAAGCGTTGTCCCCAGTG	T	80
ppiD_D2	GTCCCCAGTGGGGATGTGAC	T	70
ansB_B1	AGATCTACAAAGTTAGAGGC	NT	91
ansB_B2	TATATTTTGGAGATCTACAA	NT	81
araE_E1	TGCGACATGTCGTTATGTGA	NT	91
araE_E2	ATTAAATTGCTGCGACATGT	NT	81

[a] Template strand (T) or non-template strand (NT).

[b] Distance to TSS is the distance from the 3' end (PAM proximal) of the guide target site to the transcription start site. For synthetic promoters driven by BBa_J23117 or BBa_J23119 (http://parts.igem.org), the TSS is immediately downstream of the BBa sequence (see complete maps below).

[c] The J101-J121 sites were the same target sites used to test the positioning dependence of CRISPRa at a 10 bp resolution on the J1-J23117 promoter[3].

Supplementary Table 4. Select *E. coli* expression plasmids[a].

Plasmid	Marker	origin	Promoter	Gene	Terminator
pCD442	*CmR*	*p15A*	*1) Sp.pCas9*<br>*2) BBa_J23107*	1) dCas9<br>2) MCP-(5aa)-SoxS (R93A/S101A)	*1) BBa_B0015*<br>*2) BBa_B1002*
pCK005.1-21[b]	*CmR*	*p15A*	*1) Sp.pCas9*<br>*2) BBa_J23107*<br>*3) BBa_J23119*	1) dCas9<br>2) MCP-(5aa)-SoxS (R93A/S101A)<br>3) 1x MS2 scRNA.b2 (J101-121 targets)	*1) BBa_B0015*<br>*2) BBa_B1002*<br>*3) TrrnB*
pCD564	*CmR*	*p15A*	*1) Sp.pCas9*<br>*2) BBa_J23107*	1) dxCas9(3.7)<br>2) MCP-(5aa)-SoxS (R93A/S101A)	*1) BBa_B0015*<br>*2) BBa_B1002*
pCD565	*CmR*	*p15A*	*1) Sp.pCas9*<br>*2) BBa_J23107*<br>*3) BBa_J23119*	1) dxCas9(3.7)<br>2) MCP-(5aa)-SoxS (R93A/S101A)<br>3) 1x MS2 scRNA.b2 (J306 target)	*1) BBa_B0015*<br>*2) BBa_B1002*<br>*3) TrrnB*
pCD580.-1~-5	*AmpR*	*pSC101***	*J3_BBa_J23117 (with 1-5 bp deleted from the J1 region)*	mRFP1	*BBa_B0015*
pCD581	*CmR*	*p15A*	*1) Sp.pCas9*<br>*2) BBa_J23107*<br>*3) BBa_J23119*	1) dCas9<br>2) MCP-(5aa)-SoxS (R93A/S101A)<br>3) 1x MS2	*1) BBa_B0015*<br>*2) BBa_B1002*<br>*3) TrrnB*

				scRNA.b2<br>(J306 target)	
pJF215.x	*CmR*	*p15A*	*1) Sp.pCas9*<br>*2) TetR-pTet*<br>*3) BBa_J23119*	1) dCas9<br>2) MCP-(5aa)-SoxS (R93A/S101A)<br>3) 1x MS2 scRNA.b2 (variable target)	*1) BBa_B0015*<br>*2) BBa_B1002*<br>*3) TrrnB*
pJF076Sa[c]	*AmpR*	*pSC101***	*J3_BBa_J23117*	mRFP1	*BBa_B0015*
pJF143-J3[d]	*AmpR*	*pSC101***	*J3_BBa_J23117*	mRFP1	*BBa_B0015*
pJF155.1-12[c]	*AmpR*	*pSC101***	*J1_BBa_J23117 (with 1-12 bp inserted upstream of -35)*	mRFP1	*BBa_B0015*
pJF161.1-12[d]	*AmpR*	*pSC101***	*J3_BBa_J23117 (with 1-12 bp inserted upstream of -35)*	mRFP1	*BBa_B0015*

[a] BBa sequences are from the Repository of Standard Biological Parts (http://parts.igem.org). dCas9 is the catalytically inactive form of *S. pyogenes* Cas9. Sp.pCas9 is the endogenous Cas9 promoter from *S. pyogenes*.

[b] pCK005.1-21 indicates a set of plasmids (pCK005.1, pCK005.2…) where the final number corresponds to guide RNA target sites (J101, J102…, Supplementary Table 3) used for the J1-117-mRFP1 reporter.

[c] Originally described in previous work[3]. Modified versions of this plasmid are available where BBa_J23117 is replaced with minimal promoters regulated by alternative sigma factors (Figure 3B).

[d] Modified version of this plasmid are available where: BBa_J23117 is replaced with Anderson promoters of different strength (Figure 3A) and different PAM sites at the -81 J306 site (Figure 5A).

Supplementary Table 5. Primer sequences for RT-qPCR.

Primer	Sequence	Reference
16S_f	AAAGTTAATACCTTTGCTCATTGACGTT	[3]
16S_r	GACTACCAGGGTATCTAATCCTGTTT	[3]
aroK_f	TCTGGTTGGGCCTATGGGTG	This study
aroK_r	TACGAATGGTCACGTCGGCA	This study
cysK_f	TGCTGAAACCAGGCGTTGAA	This study
cysK_r	TCCCAACGCCAGCAATAAATACA	This study
ldhA_f	TGGCTGCGAAGCGGTATGTA	This study
ldhA_r	GAACGCCAGCAGACGCATAC	This study
yajG_fw	AAGTCACCCGCGATAAT	This study
yajG_rev	CTTTGGTCGCGATGTTG	This study
poxB_fw	GGTCTTAGTGACAGTCTTAATC	This study
poxB_r	GGAATATGAGCGGCAATC	This study

Supplementary Table 6. Intervening sequences between scRNA target site and -35 region with respective CRISPRa activity and number of transcription factor (TF) binding sites.

Index	sfGFP/OD_{600}[a]	Sequence[b]	TF binding sites, consensus[c]	TF binding sites, $d(u,v) \leq 1$[c]
1	549.87	TATCATAGTGATGACCAGTAAACATT	0	4
2	602.70	TGCAGAAAAGGGCTCTAGTGACTTGT	0	7
3	682.05	ATCTATGCGACGTCAAACGTGATGGG	0	12
4	702.47	GCCTTTCAGTGATCCCTTGTGATCTT	0	11
5	704.82	CAATTCTAGCAAGAAATTGACTTCGT	0	6
6	710.25	TTTACGGATTGGTGCGCTATTGGGTT	0	14
7	716.04	TAATGTTGATCGCCTCTATGCGTCCT	0	5
8	746.08	TTATTATAATAGTTTTGAACGTGCCT	0	6
9	794.37	CAATATGACGTGTTGTTAATTTGGTT	1	14
10	883.74	GGTCTGTGTACTCGAACACGAGATTT	0	7
11	1230.90	TTGCTCCGCGGTTTCTCTGTAAATGT	0	8
12	1532.80	ATAAATTGCATGATTAAAGACTATTG	0	11
13	1586.70	TTCGGATTTAAGGAGATATGTTTACG	0	7
14	1679.31	TAATAGGCTAGGAATTTGAAGGGATT	0	11
15	5604.77	GCATCTACATAGTGGTACAATTGAAG	0	13
16	7110.46	GCGTCCATATATGCTGATGGTGTGGG	0	10
17	7143.40	GCGGGCTATGCCTTGGAGGGTTGTAG	0	12
18	11027.55	ATTACACACGAAGGTGTATTTACTAT	2	28
19	11589.46	TTATCTAATAGGGGCGCCCCCGCTTC	0	9
20	11714.54	TTAAACAAACGAAAAGTTCGCGCCTG	1	14
21	12073.23	GATCGGGTAGGGCGCCGACAAAGGGA	0	8
22	12374.02	TGAGGGATATAGAGAGATGTTCAGCC	0	11
23	12399.33	CATCGCCGTTGCGATGCATTTTGACG	0	15
24	13207.42	GTTGTCCTTCTAGTCGCCCATGACTC	0	8
**J3**	**13269.13**	**CGTCGTCTTGAAGTTGCGATTATAGA**	0	10
25	13413.88	GTCGTAAATAAGTAAGTCACTCCCAC	2	19
26	13434.32	TTGAGGCCCATGCTTGTGGAAATGAC	1	20
27	14053.61	GGCAAGATGCCTCGTGCAGTAGAATA	0	8
28	15973.97	AGTCGCTGAGTAGATGTTCGTAGAGA	0	4
29	16760.50	ACACCGACTACCCCTGCTGGGCCCAG	0	12

[a] sfGFP/OD_{600} values represent the CRISPRa activity of strains where CRISPRa is targeted to individual elements of a reporter library where the 26 bases between the scRNA target site and the -35 region on the J3-J23117-sfGFP reporter were replaced with random bases. The

sfGFP/OD_{600} of a strain expressing the J3-J23117-sfGFP promoter are included for comparison and are in bold.

[b] The sequence corresponds to the 26 bases between the scRNA target site and the -35 region of the promoter depicted in Figure 3C.

[c] Number of exact matches to consensus transcription factor binding sites[2] or sequences within a Hamming distance $d(u,v) \leq 1$ from the consensus transcription factor binding sites.

Supplementary Methods

Computational analysis of PAM site availability

The intergenic sequences from *E. coli* were obtained from the RegulonDB database[2]. To obtain the intergenic sequences upstream of promoters, the intergenic sequences between convergent genes and between intra-operon coding sequences were removed. 5' untranslated regions (UTRs) were removed from sequences using transcription start site (TSS) information from Kim et al.[11] and RegulonDB. Intergenic sequences upstream of genes with no known TSS or where no intergenic sequence remained after removing the UTRs were discarded, yielding 1504 transcriptional units for further analysis. The number of PAM sites was calculated by counting the number of PAM sites on both strands (Supplementary Figure 10D). The PAM density over a 10 bp window was calculated by dividing the number of PAMs found at each intergenic sequence by the length of the intergenic sequence and multiplying by 10. Analyses were performed using Python 2.7.

Selection of candidate endogenous genes for CRISPRa

PAM sites found in *E. coli* intergenic sequences upstream of promoters were assigned a "sequence score" and a "distance score". The sequence score indicates the relative activity of a given PAM sequence compared to an AGG PAM, and was calculated using data from Figure 5A with dxCas9(3.7) (e.g. AGG = 1, CGT = 0.24). All NGG sequences were assigned a "sequence score" of 1. The distance score indicated the relative activity of a given PAM site position compared to a PAM site found at -81 on the non-template strand or -70 on the template from the TSS. Distance scores for the non-template strand were calculated using data from Figure 4A (e.g. -81 = 1, -91 = 0.62). For the template strand, the same scores were used, but each score was shifted upstream by 11 bases to match the position of the site of maximum activation (e.g. -70 = 1, -80 = 0.62). Each PAM site was assigned a final calculated score as "sequence score" x "position score". Promoters were then ranked by sorting for higher PAM scores at the peaks of activation (sum of the scores for the PAMs at -70, -80, -81, -91). This analysis was performed using Python 2.7. Candidates were then manually selected from among the top scoring promoters by the following criteria: (1) two or more candidate PAM sites, (2) regulation by σ^{70}, and (3) relatively weak basal expression level (<10% of the level of the maximally expressed *E. coli* gene according to data from the *E. coli* promoter collection[10]. Using these criteria, we chose the following six candidates for further characterization: *yajG*, *uxuR*, *ansB*, *poxB*, *araE*, and *ppiD*. Each of these genes is represented in the *E. coli* promoter collection (Dharmacon), a commercially available library of promoter-GFPmut2 fusions[10].

Sequence of the double mutant activation domain MCP-(5aa)-SoxS(R93A/S101A)

> MCP-(5aa)-SoxS(R93A/S101A) (optimized for minimal endogenous activity, Figure 1 and subsequent)

$MCP_{\Delta FG,\ V29I}$., 5 aa linker, SoxS(R93A/S101A) (underlined are the alanine point mutations)

MGPASNFTQFVLVDNGGTGDVTVAPSNFANGIAEWISSNSRSQAYKVTCSVRQSSAQNRKYTIKVEVPKG
AWRSYLNMELTIPIFATNSDCELIVKAMQGLLKDGNPIPSAIAANSGIYGGGGSMSHQKIIQDLIAWIDE
HIDQPLNIDVVAKKSGYSKWYLQRMFRTVTHQTLGDYIRQRRLLLAAVELRTTERPIFDIAMDLGYVSQQ
TFSRVFARQFDRTPADYRHRL

Reporter genes

The integrated sfGFP reporter, the *zwfp-lacZ* and *fumCp-lacZ* reporters used for testing the CRISPRa and endogenous activities of mutant SoxS (Figure 1) and the J1-J23117-mRFP1 reporter (Figure 3 & Supplementary Figure 5 and subsequent) for testing the distance dependence property were described in previous study[3].

J3-J23117_mRFP1 reporter (Figure 3 & Supplementary Figure 4 and subsequent)

The J3 upstream sequence contains a PAM site allowing guide RNAs to target at -81 bp to the TSS on the non-template strand, which is the same distance where maximum CRISPRa activity was observed on the J1 upstream region described previously[3].

J3 upstream region, BBa_J23117 promoter, Bujard RBS, mRFP1, BBa_B0015 terminator. The J306 target site is underlined.

AGCATTTGCGATCATTCACGCAGCGCTTATTCAGTTGCTCACTGCGATGTCATAATCATCGCTACGAGCT
GTGAAAGATGCATAAAGCTCGTACGACGCGTTCGCTCGTCTCCTCACTTCTCCTACGGAGCGTTCTGGAC
ACAACGTCGTCTTGAAGTTGCGATTATAGATTGACAGCTAGCTCAGTCCTAGGGATTGTGCTAGCGAATT
CATTAAAGAGGAGAAAGGTACCATGGCGAGTAGCGAAGACGTTATCAAAGAGTTCATGCGTTTCAAAGTT
CGTATGGAAGGTTCCGTTAACGGTCACGAGTTCGAAATCGAAGGTGAAGGTGAAGGTCGTCCGTACGAAG
GTACCCAGACCGCTAAACTGAAAGTTACCAAAGGTGGTCCGCTGCCGTTCGCTTGGGACATCCTGTCCCC
GCAGTTCCAGTACGGTTCCAAAGCTTACGTTAAACACCCGGCTGACATCCCGGACTACCTGAAACTGTCC
TTCCCGGAAGGTTTCAAATGGGAACGTGTTATGAACTTCGAAGACGGTGGTGTTGTTACCGTTACCCAGG
ACTCCTCCCTGCAAGACGGTGAGTTCATCTACAAAGTTAAACTGCGTGGTACCAACTTCCCGTCCGACGG
TCCGGTTATGCAGAAAAAAACCATGGGTTGGGAAGCTTCCACCGAACGTATGTACCCGGAAGACGGTGCT
CTGAAAGGTGAAATCAAAATGCGTCTGAAACTGAAAGACGGTGGTCACTACGACGCTGAAGTTAAAACCA

CCTACATGGCTAAAAAACCGGTTCAGCTGCCGGGTGCTTACAAAACCGACATCAAACTGGACATCACCTC
CCACAACGAAGACTACACCATCGTTGAACAGTACGAACGTGCTGAAGGTCGTCACTCCACCGGTGCTTAA
GGATCCAAACTCGAGTAAGGATCTCCAGGCATCAAATAAAACGAAAGGCTCAGTCGAAAGACTGGGCCTT
TCGTTTTATCTGTTGTTTGTCGGTGAACGCTCTCTACTAGAGTCACACTGGCTCACCTTCGGGTGGGCCT
TTCTGCGTTTATA

J3(N26)-J23117-sfGFP reporter library (Figure 3)

J3 upstream region (without 26 bases at 3’), N26, BBa_J23117 promoter, Bujard RBS, sfGFP, BBa_B0015 terminator. The J306 target site is underlined.

AGCATTTGCGATCATTCACGCAGCGCTTATTCAGTTGCTCACTGCGATGTCATAATCATCGCTACGAGCT
GTGAAAGATGCATAAAGCTCGTACGACGCGTTCGCTCGTCTCCTCACTTCTCCTACGGAGCGTTCTGGAC
ACAANNNNNNNNNNNNNNNNNNNNNNNNNNTTGACAGCTAGCTCAGTCCTAGGGATTGTGCTAGCGAATT
CATTAAAGAGGAGAAAGGTACCATGAGCAAAGGAGAAGAACTTTTCACTGGAGTTGTCCCAATTCTTGTT
GAATTAGATGGTGATGTTAATGGGCACAAATTTTCTGTCCGTGGAGAGGGTGAAGGTGATGCTACAAACG
GAAAACTCACCCTTAAATTTATTTGCACTACTGGAAAACTACCTGTTCCGTGGCCAACACTTGTCACTAC
TCTGACCTATGGTGTTCAATGCTTTTCCCGTTATCCGGATCACATGAAACGGCATGACTTTTTCAAGAGT
GCCATGCCCGAAGGTTATGTACAGGAACGCACTATATCTTTCAAAGATGACGGGACCTACAAGACGCGTG
CTGAAGTCAAGTTTGAAGGTGATACCCTTGTTAATCGTATCGAGTTAAAGGGTATTGATTTTAAAGAAGA
TGGAAACATTCTTGGACACAAACTCGAGTACAACTTTAACTCACACAATGTATACATCACGGCAGACAAA
CAAAAGAATGGAATCAAAGCTAACTTCAAAATTCGCCACAACGTTGAAGATGGTTCCGTTCAACTAGCAG
ACCATTATCAACAAAATACTCCAATTGGCGATGGCCCTGTCCTTTTACCAGACAACCATTACCTGTCGAC
ACAATCTGTCCTTTCGAAAGATCCCAACGAAAAGCGTGACCACATGGTCCTTCTTGAGTTTGTAACTGCT
GCTGGGATTACACATGGCATGGATGAGCTCTACAAATAAGGATCCAAACTCGAGTAAGGATCTCCAGGCA
TCAAATAAAACGAAAGGCTCAGTCGAAAGACTGGGCCTTTCGTTTTATCTGTTGTTTGTCGGTGAACGCT
CTCTACTAGAGTCACACTGGCTCACCTTCGGGTGGGCCTTTCTGCGTTTATA

Modified synthetic promoters

Promoters with different basal expression levels (Figure 3)

Annotations:

J3 upstream region, variable promoter (BBa_J231xx), Bujard RBS, Start codon of mRFP. The J106 target site is underlined

BBa_J23105

AGCATTTGCGATCATTCACGCAGCGCTTATTCAGTTGCTCACTGCGATGTCATAATCATCGCTACGAGCT
GTGAAAGATGCATAAAGCTCGTACGACGCGTTCGCTCGTCTCCTCACTTCTCCTACGGAGCGTTCTGGAC
ACAACGTCGTCTTGAAGTTGCGATTATAGAtttacggctagctcagtcctaggtactatgctagcGAATT
CATTAAAGAGGAGAAAGGTACCATG

BBa_J23106

AGCATTTGCGATCATTCACGCAGCGCTTATTCAGTTGCTCACTGCGATGTCATAATCATCGCTACGAGCT
GTGAAAGATGCATAAAGCTCGTACGACGCGTTCGCTCGTCTCCTCACTTCTCCTACGGAGCGTTCTGGAC
ACAACGTCGTCTTGAAGTTGCGATTATAGAtttacggctagctcagtcctaggtatagtgctagcGAATT
CATTAAAGAGGAGAAAGGTACCATG

BBa_J23107

AGCATTTGCGATCATTCACGCAGCGCTTATTCAGTTGCTCACTGCGATGTCATAATCATCGCTACGAGCT
GTGAAAGATGCATAAAGCTCGTACGACGCGTTCGCTCGTCTCCTCACTTCTCCTACGGAGCGTTCTGGAC
ACAACGTCGTCTTGAAGTTGCGATTATAGAtttacggctagctcagccctaggtattatgctagcGAATT
CATTAAAGAGGAGAAAGGTACCATG

BBa_J23108

AGCATTTGCGATCATTCACGCAGCGCTTATTCAGTTGCTCACTGCGATGTCATAATCATCGCTACGAGCT
GTGAAAGATGCATAAAGCTCGTACGACGCGTTCGCTCGTCTCCTCACTTCTCCTACGGAGCGTTCTGGAC
ACAACGTCGTCTTGAAGTTGCGATTATAGActgacagctagctcagtcctaggtataatgctagcGAATT
CATTAAAGAGGAGAAAGGTACCATG

BBa_J23109

AGCATTTGCGATCATTCACGCAGCGCTTATTCAGTTGCTCACTGCGATGTCATAATCATCGCTACGAGCT
GTGAAAGATGCATAAAGCTCGTACGACGCGTTCGCTCGTCTCCTCACTTCTCCTACGGAGCGTTCTGGAC
ACAACGTCGTCTTGAAGTTGCGATTATAGAtttacagctagctcagtcctagggactgtgctagcGAATT
CATTAAAGAGGAGAAAGGTACCATG

BBa_J23110

AGCATTTGCGATCATTCACGCAGCGCTTATTCAGTTGCTCACTGCGATGTCATAATCATCGCTACGAGCT
GTGAAAGATGCATAAAGCTCGTACGACGCGTTCGCTCGTCTCCTCACTTCTCCTACGGAGCGTTCTGGAC
ACAACGTCGTCTTGAAGTTGCGATTATAGAtttacggctagctcagtcctaggtacaatgctagcGAATT
CATTAAAGAGGAGAAAGGTACCATG

BBa_J23111

AGCATTTGCGATCATTCACGCAGCGCTTATTCAGTTGCTCACTGCGATGTCATAATCATCGCTACGAGCT
GTGAAAGATGCATAAAGCTCGTACGACGCGTTCGCTCGTCTCCTCACTTCTCCTACGGAGCGTTCTGGAC
ACAACGTCGTCTTGAAGTTGCGATTATAGAttgacggctagctcagtcctaggtatagtgctagcGAATT
CATTAAAGAGGAGAAAGGTACCATG

BBa_J23112

AGCATTTGCGATCATTCACGCAGCGCTTATTCAGTTGCTCACTGCGATGTCATAATCATCGCTACGAGCT
GTGAAAGATGCATAAAGCTCGTACGACGCGTTCGCTCGTCTCCTCACTTCTCCTACGGAGCGTTCTGGAC
ACAACGTCGTCTTGAAGTTGCGATTATAGActgatagctagctcagtcctagggattatgctagcGAATT
CATTAAAGAGGAGAAAGGTACCATG

BBa_J23113

AGCATTTGCGATCATTCACGCAGCGCTTATTCAGTTGCTCACTGCGATGTCATAATCATCGCTACGAGCT
GTGAAAGATGCATAAAGCTCGTACGACGCGTTCGCTCGTCTCCTCACTTCTCCTACGGAGCGTTCTGGAC
ACAACGTCGTCTTGAAGTTGCGATTATAGActgatggctagctcagtcctagggattatgctagcGAATT
CATTAAAGAGGAGAAAGGTACCATG

BBa_J23114

AGCATTTGCGATCATTCACGCAGCGCTTATTCAGTTGCTCACTGCGATGTCATAATCATCGCTACGAGCT
GTGAAAGATGCATAAAGCTCGTACGACGCGTTCGCTCGTCTCCTCACTTCTCCTACGGAGCGTTCTGGAC
ACAACGTCGTCTTGAAGTTGCGATTATAGAtttatggctagctcagtcctaggtacaatgctagcGAATT
CATTAAAGAGGAGAAAGGTACCATG

BBa_J23115

AGCATTTGCGATCATTCACGCAGCGCTTATTCAGTTGCTCACTGCGATGTCATAATCATCGCTACGAGCT
GTGAAAGATGCATAAAGCTCGTACGACGCGTTCGCTCGTCTCCTCACTTCTCCTACGGAGCGTTCTGGAC
ACAACGTCGTCTTGAAGTTGCGATTATAGAtttatagctagctcagcccttggtacaatgctagcGAATT
CATTAAAGAGGAGAAAGGTACCATG

BBa_J23118

AGCATTTGCGATCATTCACGCAGCGCTTATTCAGTTGCTCACTGCGATGTCATAATCATCGCTACGAGCT
GTGAAAGATGCATAAAGCTCGTACGACGCGTTCGCTCGTCTCCTCACTTCTCCTACGGAGCGTTCTGGAC

ACAACGTCGTCTTGAAGTTGCGATTATAGAttgacggctagctcagtcctaggtattgtgctagcGAATT
CATTAAAGAGGAGAAAGGTACCATG

BBa_J23119
AGCATTTGCGATCATTCACGCAGCGCTTATTCAGTTGCTCACTGCGATGTCATAATCATCGCTACGAGCT
GTGAAAGATGCATAAAGCTCGTACGACGCGTTCGCTCGTCTCCTCACTTCTCCTACGGAGCGTTCTGGAC
ACAACGTCGTCTTGAAGTTGCGATTATAGAttgacagctagctcagtcctaggtataatgctagcGAATT
CATTAAAGAGGAGAAAGGTACCATG

Promoters regulated by alternative sigma factors (Figure 3)

Annotations:

J1 upstream region, variable promoter (color coded), Bujard RBS, Start codon of mRFP. The J106 target site is underlined.

J1-*sodCp* promoter
GCCTACGGTATCCACCGGAGACCTATGGCAGCCTCCGGCCGCCATAGGACACCTTTGGTTGCCAAGGGTG
ACCTATGGTGACCATGGGCCACCACGGGCGACCTCAGGTATCCTGCGGTGTCCTGCGGTTACCAAAGGCG
TCCTTTGGGTTCCACCGGATACCTCCGGCCGTTcaaaaatgtgtcacTGGTTTACACTtattcagGGAAT
TCATTAAAGAGGAGAAAGGTACCATG

In the J1-*sodCp* promoter, we deleted 3 bp between the J1 sequence and the *sodCp* minimal promoter to maintain the same -80 bp spacing between the target site and the J109 target site as in the rpoD promoter. Without this deletion, activation from the *sodCp* promoter is substantially weaker (not shown), likely due to the sensitive positioning requirements described in Figure 4.

J1-*glnAp2* promoter
GCCTACGGTATCCACCGGAGACCTATGGCAGCCTCCGGCCGCCATAGGACACCTTTGGTTGCCAAGGGTG
ACCTATGGTGACCATGGGCCACCACGGGCGACCTCAGGTATCCTGCGGTGTCCTGCGGTTACCAAAGGCG
TCCTTTGGGTTCCACCGGATACCTCCGGACTGGCACagatttCGCTTtatcttttTacggcgacGAATT
CATTAAAGAGGAGAAAGGTACCATG

J1-*rdgBp* promoter
GCCTACGGTATCCACCGGAGACCTATGGCAGCCTCCGGCCGCCATAGGACACCTTTGGTTGCCAAGGGTG
ACCTATGGTGACCATGGGCCACCACGGGCGACCTCAGGTATCCTGCGGTGTCCTGCGGTTACCAAAGGCG

TCCTTTGGGTTCCACCGGATACCTCCGGACTTGTAAaaggcgaacttggcCGCCACaaacaaattGAATT
CATTAAAGAGGAGAAAGGTACCATG

J1-*yieEp* promoter
GCCTACGGTATCCACCGGAGACCTATGGCAGCCTCCGGCCGCCATAGGACACCTTTGGTTGCCAAGGGTG
ACCTATGGTGACCATGGGCCACCACGGGCGACCTCAGGTATCCTGCGGTGTCCTGCGGTTACCAAAGGCG
TCCTTTGGGTTCCACCGGATACCTCCGGACGAACTTttagccgctttagtctgtcCATCAttccAGAATT
CATTAAAGAGGAGAAAGGTACCATG

J3(tetO)-J23117_mRFP1 reporter (Figure 3)
J3 upstream region (without 19 bases at 3’), tetO, BBa_J23117 promoter, Bujard RBS, ATG of mRFP1. The J306 target site is underlined.

AGCATTTGCGATCATTCACGCAGCGCTTATTCAGTTGCTCACTGCGATGTCATAATCATCGCTACGAGCT
GTGAAAGATGCATAAAGCTCGTACGACGCGTTCGCTCGTCTCCTCACTTCTCCTACGGAGCGTTCTGGAC
ACAACGTCGTCACTCTATCGTTGATAGAGTttgacagctagctcagtcctagggattgtgctagcGAATT
CATTAAAGAGGAGAAAGGTACCATG

J1-J23117 promoter with shifted bases (Figure 4, Supplementary Figure 5 and subsequent)
For promoters shifted by fewer than 12bp, bases were removed from the 5’ end of the inserted sequence (starting with C).
J1 upstream region, inserted sequence (12 bases), BBa_J23117 promoter, Bujard RBS, ATG of mRFP1. The J106 target site is underlined.

GCCTACGGTATCCACCGGAGACCTATGGCAGCCTCCGGCCGCCATAGGACACCTTTGGTTGCCAAGGGTG
ACCTATGGTGACCATGGGCCACCACGGGCGACCTCAGGTATCCTGCGGTGTCCTGCGGTTACCAAAGGCG
TCCTTTGGGTTCCACCGGATACCTCCGGACCTAGATGCGCTCttgacagctagctcagtcctagggattg
tgctagcGAATTCATTAAAGAGGAGAAAGGTACCATG

J3-J23117 promoter with shifted bases (Supplementary Figure 5 and subsequent)
For promoters shifted by fewer than 12bp, bases were removed from the 5’ end of the inserted sequence (starting with T). For promoters with negative shifts, no sequence was inserted between the J3 upstream region and BBa_J23117, and bases were removed from the 3’ end of the J3 upstream region (starting with A).

J3 upstream region, inserted sequence (12 bases), BBa_J23117 promoter, Bujard RBS, ATG of mRFP1. The J306 target site is underlined.

AGCATTTGCGATCATTCACGCAGCGCTTATTCAGTTGCTCACTGCGATGTCATAATCATCGCTACGAGCT
GTGAAAGATGCATAAAGCTCGTACGACGCGTTCGCTCGTCTCCTCACTTCTCCTACGGAGCGTTCTGGAC
ACAACGTCGTCTTGAAGTTGCGATTATAGATGAGATGCGCTCttgacagctagctcagtcctagggattg
tgctagcGAATTCATTAAAGAGGAGAAAGGTACCATG

Modified J3-J23117 promoter with 6 adjacent PAMs around -81 bp to TSS (Supplementary Figure 5)
J3 upstream region, BBa_J23117 promoter, Bujard RBS, Start codon of mRFP. Region with additional PAM sites inserted. The J306 target site is underlined.

AGCATTTGCGATCATTCACGCAGCGCTTATTCAGTTGCTCACTGCGATGTCATAATCATCGCTACGAGCT
GTGAAAGATGCATAAAGCTCGTACGACGCGTTCGCTCGTCTCCTCACCCCCCCTACGGAGCGTTCTGGAC
ACAACGTCGTCTTGAAGTTGCGATTATAGAttgacagctagctcagtcctagggattgtgctagcGAATT
CATTAAAGAGGAGAAAGGTACCATG

Modified J3-J23117 promoter with non-NGG PAMs around -81 bp to TSS (Figure 5A)
J3 upstream region, BBa_J23117 promoter, Bujard RBS, Start codon of mRFP, region with modified PAMs. The J306 target site is underlined.

AGCATTTGCGATCATTCACGCAGCGCTTATTCAGTTGCTCACTGCGATGTCATAATCATCGCTACGAGCT
GTGAAAGATGCATAAAGCTCGTACGACGCGTTCGCTCGTCTCCTCACTTCTNNNACGGAGCGTTCTGGAC
ACAACGTCGTCTTGAAGTTGCGATTATAGAttgacagctagctcagtcctagggattgtgctagcGAATT
CATTAAAGAGGAGAAAGGTACCATG

Promoter to demonstrate dxCas9(3.7) versatility (Figure 5B)
J3 upstream region, BBa_J23117 promoter, Bujard RBS, Start codon of mRFP. The modified target site (M) is underlined. AGT PAM site.

AGCATTTGCGATCATTCACGCAGCGCTTATTCAGTTGCTCACTGCGATGTCATAATCATCGCTACGAGCT
GTGAAAGATGCATAAAGCTCGTACGACGCGTTCGCTCGTCTCCTCACTTCTCCTACACTGCTGACACTTC

TGCTCGTCGTCTTGAAGTTGCGATTATAGAttgacagctagctcagtcctagggattgtgctagcGAATT
CATTAAAGAGGAGAAAGGTACCATG

Supplementary References

1. Keseler, I. M. *et al.* The EcoCyc database: reflecting new knowledge about *Escherichia coli* K-12. *Nucleic Acids Res* **45**, D543–D550 (2017).
2. Gama-Castro, S. *et al.* RegulonDB version 9.0: high-level integration of gene regulation, coexpression, motif clustering and beyond. *Nucleic Acids Res* **44**, D133–D143 (2016).
3. Dong, C., Fontana, J., Patel, A., Carothers, J. M. & Zalatan, J. G. Synthetic CRISPR-Cas gene activators for transcriptional reprogramming in bacteria. *Nat Commun* **9**, 2489 (2018).
4. Griffith, K. L. & Wolf, R. E. A comprehensive alanine scanning mutagenesis of the *Escherichia coli* transcriptional activator SoxS: Identifying amino acids important for DNA binding and transcription activation. *Journal of Molecular Biology* **322**, 237–257 (2002).
5. Ryoichi Arai, T. N., Hiroshi Ueda, Atsushi Kitayama, Noriho Kamiya. Design of the linkers which effectively separate domains of a bifunctional fusion protein. *Protein Engineering, Design and Selection* **14**, 529–532 (2001).
6. Zalatan, J. G. *et al.* Engineering complex synthetic transcriptional programs with CRISPR RNA scaffolds. *Cell* **160**, 339–350 (2015).
7. Konermann, S. *et al.* Genome-scale transcriptional activation by an engineered CRISPR-Cas9 complex. *Nature* **517**, 583–588 (2015).
8. Bikard, D. *et al.* Programmable repression and activation of bacterial gene expression using an engineered CRISPR-Cas system. *Nucleic Acids Res* **41**, 7429–7437 (2013).
9. Johnny H. Hu, D. R. L., Shannon M. Miller, Maarten H. Geurts, Weixin Tang, Liwei Chen, Ning Sun, Christina M. Zeina, Xue Gao, Holly A. Rees, Zhi Lin. Evolved Cas9 variants with broad PAM compatibility and high DNA specificity. *Nature* **556**, 57–63 (2018).

10. Zaslaver, A. *et al.* A comprehensive library of fluorescent transcriptional reporters for *Escherichia coli*. *Nat Methods* **3**, 623–628 (2006).

11. Donghyuk Kim, B. Ø. P., Jay Sung-Joong Hong, Yu Qiu, Harish Nagarajan, Joo-Hyun Seo, Byung-Kwan Cho, Shih-Feng Tsai. Comparative analysis of regulatory elements between *Escherichia coli* and *Klebsiella pneumoniae* by genome-wide transcription start site profiling. *PLoS Genetics* **8**, e1002867 (2012).
