# Supplementary material for: Effective CRISPRa-mediated control of gene expression in bacteria must overcome strict target site requirements
Source: Nat Commun. 2020 Apr 1;11:1618. doi: 10.1038/s41467-020-15454-y (PMC7113249; doi:10.1038/s41467-020-15454-y)
Supplement: Supplementary file 2 — Reporting Summary [file 41467_2020_15454_MOESM2_ESM.pdf]

nature research

Corresponding author(s): James Carothers, Jesse Zalatan

Last updated by author(s): Feb 24, 2020

Reporting Summary

Nature Research wishes to improve the reproducibility of the work that we publish. This form provides structure for consistency and transparency in reporting. For further information on Nature Research policies, see Authors & Referees and the Editorial Policy Checklist .

Statistics

For all statistical analyses, confirm that the following items are present in the figure legend, table legend, main text, or Methods section.

n/a	Confirmed	
☐	☒	The exact sample size (*n*) for each experimental group/condition, given as a discrete number and unit of measurement
☐	☒	A statement on whether measurements were taken from distinct samples or whether the same sample was measured repeatedly
☐	☒	The statistical test(s) used AND whether they are one- or two-sided<br>*Only common tests should be described solely by name; describe more complex techniques in the Methods section.*
☐	☒	A description of all covariates tested
☒	☐	A description of any assumptions or corrections, such as tests of normality and adjustment for multiple comparisons
☐	☒	A full description of the statistical parameters including central tendency (e.g. means) or other basic estimates (e.g. regression coefficient) AND variation (e.g. standard deviation) or associated estimates of uncertainty (e.g. confidence intervals)
☐	☒	For null hypothesis testing, the test statistic (e.g. *F*, *t*, *r*) with confidence intervals, effect sizes, degrees of freedom and *P* value noted<br>*Give P values as exact values whenever suitable.*
☒	☐	For Bayesian analysis, information on the choice of priors and Markov chain Monte Carlo settings
☒	☐	For hierarchical and complex designs, identification of the appropriate level for tests and full reporting of outcomes
☒	☐	Estimates of effect sizes (e.g. Cohen's *d*, Pearson's *r*), indicating how they were calculated

Our web collection on statistics for biologists contains articles on many of the points above.

Software and code

Policy information about availability of computer code

Data collection	Plate reader data was collected using BioTek Gen5 2.07.17. Flow cytometry data was collected using MACSQuantify 2.8. RT-qPCR data was collected using Bio-Rad CFX Maestro 4.0.23225.0418.
Data analysis	Fold change values in RT-qPCR data were calculated using Bio-Rad CFX Maestro 4.0.23225.0418. Median fluorescence in flow cytometry data was calculated using FlowJo 10.0.7. Microsoft Excel 16.29 was used for data and statistical analysis. Custom code based on Python 2.7 was used for calculating the density of PAM sites between transcriptional units in E. coli.

For manuscripts utilizing custom algorithms or software that are central to the research but not yet described in published literature, software must be made available to editors/reviewers. We strongly encourage code deposition in a community repository (e.g. GitHub). See the Nature Research guidelines for submitting code & software for further information.

Data

Policy information about availability of data

All manuscripts must include a data availability statement. This statement should provide the following information, where applicable:

- Accession codes, unique identifiers, or web links for publicly available datasets
- A list of figures that have associated raw data
- A description of any restrictions on data availability

All data from this study are available upon request.

Field-specific reporting

Please select the one below that is the best fit for your research. If you are not sure, read the appropriate sections before making your selection.

October 2018

Life sciences study design

All studies must disclose on these points even when the disclosure is negative.

Sample size	Sample sizes were determined following standard practice in the field (Qi et al, 2013 doi: 10.1016/j.cell.2013.02.022; Bikard et al., 2013 doi: 10.1093/nar/gkt520; Dong et al., 2018 doi: 10.1038/s41467-018-04901-6). No statistical tests were used to determine sample size.
Data exclusions	In Figure 4A, 1 of 3 biological replicates in 4 data points (J104 at -81, -82, -83; J108 at -102) were excluded because cultures failed to grow.
Replication	Experiments were performed in triplicate to ensure reproducibility between biological replicates, except in the following cases which are also noted in the figure legends:<br>Supplementary Figure 1: three technical replicates of one biological replicate were performed.<br>Supplementary Figure 11: two biological replicates were performed for the yajG sample and 4 biological replicates were performed for the poxB sample.
Randomization	Biological replicates were obtained from cultures inoculated from single colonies chosen randomly from agar plates.
Blinding	The investigators were not blinded as the data collected in this work did not require a subjective analysis by the experimenters.

Reporting for specific materials, systems and methods

We require information from authors about some types of materials, experimental systems and methods used in many studies. Here, indicate whether each material, system or method listed is relevant to your study. If you are not sure if a list item applies to your research, read the appropriate section before selecting a response.

Materials & experimental systems

n/a	Involved in the study
☒	☐ Antibodies
☒	☐ Eukaryotic cell lines
☒	☐ Palaeontology
☒	☐ Animals and other organisms
☒	☐ Human research participants
☒	☐ Clinical data

Methods

n/a	Involved in the study
☒	☐ ChIP-seq
☐	☒ Flow cytometry
☒	☐ MRI-based neuroimaging

Flow Cytometry

Plots

Confirm that:

☐ The axis labels state the marker and fluorochrome used (e.g. CD4-FITC).

☒ The axis scales are clearly visible. Include numbers along axes only for bottom left plot of group (a 'group' is an analysis of identical markers).

☐ All plots are contour plots with outliers or pseudocolor plots.

☐ A numerical value for number of cells or percentage (with statistics) is provided.

Methodology

Sample preparation	E. coli cells from overnight cultures were diluted 1:50 in DPBS before analysis on the flow cytometer.
Instrument	MACSQuant VYB flow cytometer
Software	Collection: MACSQuantify 2.8; Analysis: FlowJo 10.0.7
Cell population abundance	Bacterial cell populations were gated for single cells as described below. 10,000 events were collected per sample.
Gating strategy	A figure exemplifying the gating strategy is available in our prior work (doi: 10.1038/s41467-018-04901-6).<br><br>A side scatter threshold trigger (SSC-H) was applied to enrich for single cells. A narrow gate along the diagonal line on the SSC-H vs SSC-A plot was selected to exclude the events where multiple cells were grouped together. Within the selected population, events that appeared on the edges of the FSC-A vs. SSC-A plot and the fluorescence histogram were excluded.

☐ Tick this box to confirm that a figure exemplifying the gating strategy is provided in the Supplementary Information.
